# miR-223 Inhibits the Polarization and Recruitment of Macrophages via NLRP3/IL-1*β* Pathway to Meliorate Neuropathic Pain

**DOI:** 10.1155/2021/6674028

**Published:** 2021-08-06

**Authors:** Junsong Zhu, Jinmei Yang, Jianguo Xu

**Affiliations:** ^1^Pain Department, Puren Hospital Affiliated to Wuhan University of Science and Technology, Wuhan 430081, China; ^2^Wuhan Hospital of Traditional Chinese and Western Medicine, Wuhan 430022, China; ^3^Hubei Provincial Hospital of Traditional Chinese Medicine, Wuhan 430061, China; ^4^Hubei Provincial Academy of Traditional Chinese Medicine, Wuhan 430074, China

## Abstract

**Background:**

miRNA is an essential factor in neuropathic pain. However, the underlying mechanism of miRNA in neuropathic pain remains unclear.

**Objective:**

To explore the potential role of miR-223 in neuropathic pain in a mice model of chronic sciatic nerve injury.

**Methods:**

Mice were divided into the sham group, CCI group, CCI + Lenti-vector group, and CCI + Lenti-miR-223 group. Flow cytometry was used to detect the neuronal apoptosis and the proportion of M1/M2 macrophages in each group. Western blot was used to detect the protein expression levels of ASC, caspase-1, IL-1*β*, and IL-18 in each group. Luciferase activity assay detects the binding of miR-223 and NLRP3. Macrophage chemotaxis experiments verified the anti-inflammatory effect of miR-223 in vitro.

**Results:**

The overexpression of miR-233 significantly reduced the neuropathic pain caused by CCI and reduced the apoptosis and inflammatory factor expression. miR-223 inhibits the expression of NLRP3 by directly binding to the 3′-untranslated region. Overexpression of miR-223 reduces the protein levels of NLRP3, ASC, caspase-1, IL-1*β*, and IL-18 in the spinal cord of CCI mice, increases the proportion of M2-type macrophages, and reduces the proportion of M1-type macrophages.

**Conclusion:**

miR-223 may facilitate the development of neuropathic pain in CCI mice by inhibiting NLRP3-mediated neuroinflammation.

## 1. Introduction

Neuropathic pain refers to pain caused by the damage to the somatosensory system or disease. It is estimated that its prevalence may be between 6.9% and 10% in the general population [1]. There are millions of patients worldwide who do not have an exact cause and treatment method. The most common causes include poisoning, surgery, radiation, trauma, and congenital diseases [2]. Due to the damage to the nervous system, neuropathic pain is defined as allodynia and hyperalgesia, which can respond to painless or harmful stimuli [3]. Neuropathic pain can lead to pathological pain, which will persist for an extended period. But, the molecular mechanisms of neuropathic pain remain poorly known.

miRNA is a subset of endogenous single-stranded noncoding small RNAs, ranging in size from 17 to 21 nucleotides [4]. At present, miRNA's role in neuropathic pain has received more and more attention in the medical field, and related studies have confirmed the changes in miRNA expression in neuropathic pain [5, 6]. Recently, it has been reported that miR-223 has become a key regulator of bacterial stimulation and immune system response. miR-223 is transcribed from an independent promoter and expressed specifically in the hematopoietic system [7]. Also, miR-223 plays a crucial role in inflammatory diseases by regulating different gene transcription factors (including C/EBPa, E2F1, and NF-*κ*B pathway) [8]. Recently, it was discovered that NLRP3 is one of the target genes of miR-223. Studies have indicated that miR-223 negatively regulates NLRP3, thereby promoting the secretion of certain macrophages [9].

A large number of studies indicate that the occurrence and maintenance of neuropathic pain depend on the interaction with immune cells of sensory neurons and the activation of immune receptors expressed by sensory neurons. The main sensitization signal transduction pathway involves the proinflammatory cytokine interleukin-1*β* (IL-1*β*). It is known that IL-1*β* causes pain by directly acting on sensory neurons [10]. There is evidence that blocking IL-1*β* signaling can reduce postoperative pain, but completely blocking IL-1*β* signaling can increase infection risk and reduce effective wound healing [11]. IL-1*β* requires activation by an inflammasome; inflammasomes are cytosolic receptors of the innate immune system. The Nod-like receptor protein 3 (NLRP3), a member of NLRs family NLRP3s, consisting of three main proteins, including NLRP3 scaffold, regulatory molecule caspase-1, and apoptosis-associated speck-like protein containing a CARD (ASC), has emerged as a crucial regulator of chronic inflammatory disease [12, 13]. It mediated the activation of caspase-1 in response to microbial ligands and then cleaved and activated pro-IL-1*β* and pro-IL-18 to active forms and promoted their secretion [14]. However, little is known about the role of miR-223 and NLRP3 in neuropathic pain and aseptic inflammation. Therefore, our study aims to investigate the effects of miR-223 on ameliorating neuropathic pain by targeting NLRP3 in the mice model.

## 2. Materials and Methods

### 2.1. Construction of the Neuropathic Pain Model

All experimental animal procedures were according to the guidelines of the International Association for Pain and under the Guide for the Care and Use of Laboratory Animals of the NIH. The ethics committee approved the study in Puren Hospital Affiliated with Wuhan University of Science and Technology. A bilateral CCI mice model was used. Intraperitoneal injection of sodium pentobarbital (40–50 mg/kg) was used. The sciatic nerve was exposed using blunt dissection. Sciatic nerves were tied, and the nerve's length was 4-5 mm long proximal to the sciatic's trifurcation. After washed with physiological saline, the incision was closed. For another, an identical dissection was carried out on the other side. Mice were maintained in plastic cages with solid floors. Via an intrathecal catheter, recombinant lentivirus (GenePharma, Shanghai, China), Lenti-vector, and Lenti-miR-223 were injected through a microinjection syringe. Lenti-vector and Lenti-miR-223 were injected for one time three days before surgery. Ten microliters of recombinant lentivirus at a multiplicity of infection (MOI) of 20 were injected.

### 2.2. Cell Culture

Mice microglial cells and BV2 were acquired from Science (Carlsbad, CA). The cells were cultured in Dulbecco's modified Eagles medium (DMEM) with 10% HyClone, 100 IU/ml penicillin, and 100 *μ*g/ml streptomycin (Sigma, America) with 5% CO_2_ at 37°C.

### 2.3. Thermal Hyperalgesia and Mechanical Allodynia Evaluation

Thermal hyperalgesia is measured by the incubation period of pain relief. Place the mice in a transparent plexiglass compartment and adapt to the environment. The radiant heat of constant intensity (beam diameter 0.5 cm, intensity 20 I.R.) was applied to the midfoot area of the hind paw until the animal lifted its foot from the floor. The time from initial heating to paw extraction was recorded.

Mechanical allodynia was evaluated by the paw mechanical withdrawal threshold measured with a dynamic plantar anesthesia instrument, and the mice were placed in a test cage with a wire mesh floor and adapted. Apply the hard tip of the filament to the midfoot area of the hind paw with greater force (maximum 5 g in 20 s) until the animal removes its foot. The average value of the withdrawal threshold force was calculated from four independent experiments.

### 2.4. Enzyme-Linked Immunosorbent Assay (ELISA)

On the 7th postoperative day, spinal cords of sham mice (*n* = 6), CCI mice (*n* = 6), and CCI mice were infected with Lenti-miR-223 (*n* = 6) or Lenti-vector (*n* = 6). The tissue was taken out from the −80°C refrigerator and ground to extract protein. The protein expression of TNF-*α* and IL-1*β* was measured according to the manufacturer's instructions using a commercially available ELISA kit (R&D Systems).

### 2.5. Flow Cytometry Assay

The mice nerve tissue was cut into small pieces and placed in a buffer containing RPMI, collagenase IV (Sigma, America), and DNase I (Sigma, America) and then incubated at 37°C for 1 hour. Use a 70 lm filter to remove debris and then centrifuge. The sample was incubated with a solution containing 2.4G2 antibody at 4°C for 30 minutes. Then, the cell surface antigen was stained with a specific fluorescent dye-conjugated rat anti-mice antibody for 30 minutes at 4°C. After staining the cell surface antigen, the sample was fixed with 4% PFA at 4°C for 15 minutes and then permeabilized using an immobilization/permeation kit (Abcam) for 30 minutes. It was then resuspended in 1X Perm/wash solution containing antibody at 1°C for 30 minutes and suspended at 4°C. The staining specificity was verified with the prepared control (without primary antibody), and spectral overlap correction was performed by using negative and positive compensation beads, and the data were analyzed using FlowJo software. Different combinations of antibodies are used in CD45, CD11b, CD68, CD86, and CD206 and Annexin V-FITC/PI apoptosis detection kits. Use FlowJo software to analyze flow cytometer data.

### 2.6. Western Blot

Spinal cord tissues of sham mice (*n* = 6), CCI mice (*n* = 6), and CCI mice infected with Lenti-miR-223 (*n* = 6) or Lenti-vector (*n* = 6) were buffered with RIPA. The solution is lysed, and the total protein is extracted. The separation of proteins was achieved by 10% SDS-PAGE, and then, the separated proteins were transferred to PVDF membranes. Incubate the membrane with 5% milk at room temperature for 1–1.5 hours and then incubate with the primary antibody at 4°C overnight. The primary antibodies are anti-NLPR3, anticaspase-1 cleavage, anti-ASC, anti-IL-1*β*, anti-IL-18, and anti-*β*-actin (both from Abcam). The blot was washed again with Tris buffered saline/Tween 20 (TBST) 3 times and then incubated with a secondary antibody diluted 1 : 5000 at room temperature for 1 hour. The blot was washed 3 times with TBST again and then developed by enhanced chemiluminescence. The band intensity was quantified using UN-SCAN-IT gel analysis software, and the protein expression was normalized to the expression of *β*-actin.

### 2.7. Real-Time PCR

Total RNA of lumbar spinal segment of the designated mice (*n* = 6 per group) was extracted with TRIzol reagent. According to the manufacturer's instructions, miRNA cDNA was synthesized using the Mir-X miRNA first-strand synthesis kit. The primer sequences are as follows: miR-223: forward, 5′-GCGCGTGTCAGTTTGTCAAAT-3′, reverse, 5′-AGTGCAGGGTCCGAGGTATT-3'; U6: forward 5′-CTCGCTTCGGCAGCACA-3′, reverse 5′- AACGCTTCACGAATTTGCGT-3′. After incubation at 95°C for 15 minutes, qRT-PCR was performed at 95°C for 40 cycles of 15 s and at 60°C for 1 minute. The expression of miR-223 was normalized to U6.

### 2.8. Macrophage Chemotaxis Experiment

Primary microglial cells were prepared from mice injected with Lenti-vector or Lenti-miR-223 and seeded in 24-well plates [15]. When microglia reached 80–90% confluence, they were replaced with normal medium after treatment with 100 ng/ml LPS for 3 h. Then, transwell was used to coculture RAW264.7 in the upper chamber. After 72 h of culture, the transwell was taken out, stained, and the number of RAW264.7 cells entering the lower chamber was counted.

### 2.9. Luciferase Assay

A luciferase-3′-UTR reporter gene construct was designed using NLRP3 mRNA with a putative miR-223 binding site. Construct a plasmid vector with wild-type (wt) NLRP3 and a plasmid vector with mutant (mut) NLRP3. BV2 cells were seeded in a 12-well plate at a concentration of 1 × 105 cells/mL and then mixed with wild-type (wt) or mutant (mut) for total transfection. Lipofectamine 2000 transfection kit was used to transfect PGL3-NLRP3 3′-UTR and miR-223 mimic and control. After 48 hours, the Infinite 200 PRO series luminometer (Thermo Fisher, Massachusetts, USA) was used to test the luciferase activity, and data are expressed as the ratio of the measured luciferase activity to the control.

### 2.10. Statistical Analyses

Data were from three independent experiments and expressed as mean ± SEM. Statistical analyses were performed by the one-way analysis of variance (ANOVA) for differences among different groups. All analyses were undertaken using GraphPad Prism 6. *P* < 0.05 and *P* < 0.01 were considered statistically significant.

## 3. Results

### 3.1. Overexpressed miR-223 Alleviates CCI-Induced Neuropathic Pain and Tissue Damage

**Figure 1:**
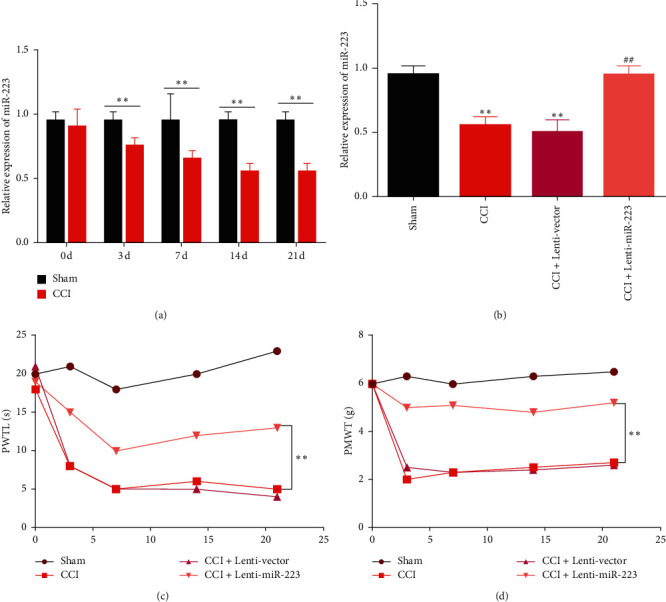
Overexpression of miR-223 alleviates CCI-induced neuropathic pain and tissue damage. (a) miR-223 levels in the spinal cords of mice were measured by qRT-PCR at postoperative days 0, 3, 7, 14, and 21. *n* = 6 for each time point. ^*∗∗*^*P* < 0.01 compared to the sham group. (b) The expression of miR-223 in the spinal cords of CCI model mice infected with lentiviral vectors carrying miR-223 (Lenti-miR-223) or lentiviral vector (Lenti-vector) at postoperative day 7. *n* = 6 per group. ^*∗∗*^*P* < 0.01 compared to the sham group. ^##^*P* < 0.01 compared to the CCI group. (c) The effect of miR-223 overexpression on thermal hyperalgesia was assessed by paw withdrawal thermal latency (PWTL). *n* = 6 per group. ^*∗∗*^*P* < 0.01. (d) The effect of miR-223 upregulation on mechanical allodynia was evaluated according to the paw mechanical withdrawal threshold (PMWT). *n* = 6 per group. ^*∗∗*^*P* < 0.01.

In order to study the relationship between miR-223 and neuropathic pain, mice with chronic sciatic nerve contraction were produced by surgical ligation, and the expression of miR-223 in the spinal cord was measured at 0, 3, 7, 14, and 0, respectively, 21 days after CCI. The spinal cord of the mice was then collected and analyzed for miR-223 levels in the spinal cord by qRT-PCR analysis. The data showed that in CCI mice, miR-223 was significantly downregulated compared to mice undergoing sham surgery (Figure 1(a)). To further study the biological role of miR-223 in neuropathic pain, miR-223 was overexpressed in mice by intrathecal injection of lentivirus to express miR-223. In the injected mice, miR-223 was stably overexpressed compared to the CCI group (Figure 1(b)). In addition, our results indicate that miR-223 overexpression attenuates CCI-induced mechanical allodynia (Figure 1(c)) and thermal hyperalgesia (Figure 1(d)). Neuropathological behaviors such as mechanical allodynia and thermal hyperalgesia are significantly reduced due to miR-223 overexpression, which implies the positive role of miR-223 in neuropathic pain.

### 3.2. Overexpression of miR-223 Inhibits Cell Apoptosis and Neuroinflammation in CCI

**Figure 2:**
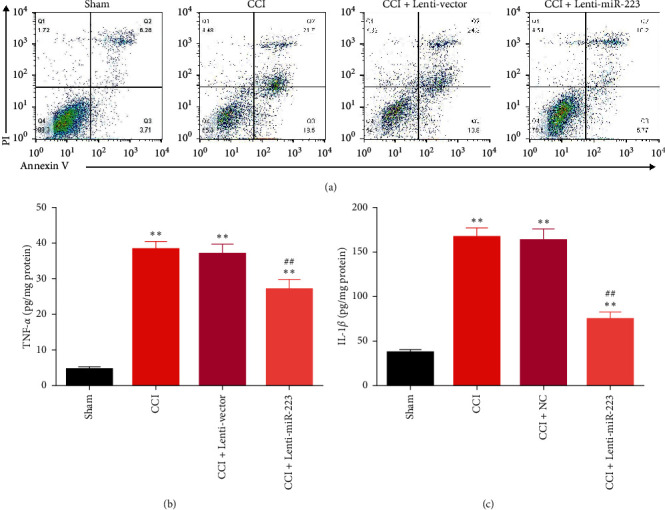
Overexpression of miR-223 inhibits cell apoptotic and neuroinflammation in CCI mice. (a) Flow cytometry assays were performed on the spinal cord sections from each group at day 7 after CCI. The spinal cord tissue levels of TNF-*α* (b) and IL-1*β* (c) were measured by ELISA in all groups. *n* = 6. ^*∗∗*^*P* < 0.01 compared to the sham group. ^##^*P* < 0.05 compared to the CCI group.

To further study the biological effects of miR-223 on apoptosis and neuroinflammation, flow cytometry was performed on the collected spinal cord sections on the 7th day after CCI. The proportion of apoptotic cells in CCI mice was significantly higher than in sham-operated mice (Figure 2(a)). To assess the level of neuroinflammation, ELISA was performed to detect the levels of TNF-*α* and IL-1*β* 7 days after CCI. The data showed that the levels of TNF-*α* (Figure 2(b)) and IL-1*β* (Figure 2(c)) were upregulated in all CCI treatment groups. Compared with the CCI group, overexpression of miR-223 significantly prevented apoptosis and neuroinflammation level. The upregulation caused by CCI indicates that miR-223 may prevent apoptosis and inflammation in CCI mice.

### 3.3. miR-223 Could Ameliorate Neuroinflammation via Targeting NLRP3

**Figure 3:**
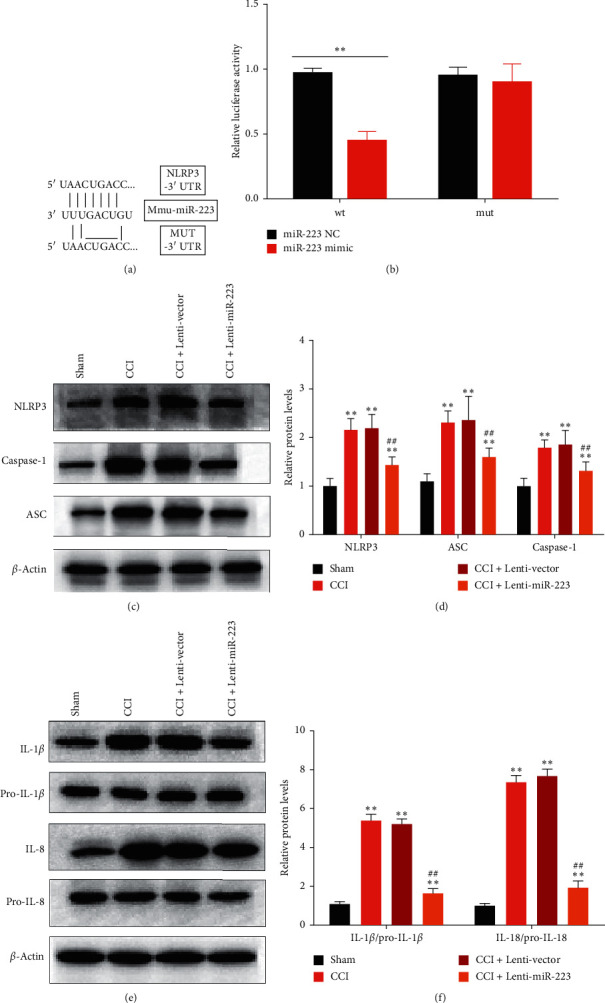
MiR-223 could ameliorate neuroinflammation via targeting NLRP3. (a) Schematic representation of the predicted binding sites between miR-223 and NLRP3. (b) Dual-luciferase reporter assays of miR-223 and NLRP3 3′-UTR. The miR-223 mimics or the control (miR-223 NC) was cotransfected with the dual-luciferase vector containing wt or mut pGL3-NLRP3 3′-UTR into BV2 cells and incubated for 48 h. The relative luciferase activity was calculated as the ratio of firefly to Renilla luciferase activity, as measured with the dual-luciferase assay system. ^*∗∗*^*P* < 0.01 compared to the miR-223 NC group. (c) Western blot detecting the expression of NLRP3, ASC, and activated caspase-1 in the spinal cords of CCI model mice infected with Lenti-vector or with Lenti-miR-223. (d) Quantification of NLRP3, ASC, and caspase-1 expression levels; data were normalized by *β*-actin. *n* = 6. ^*∗∗*^*P* < 0.01 compared to the sham group. ^##^*P* < 0.05 compared to the CCI group. (e) Representative picture showing the Western blot analysis for IL-1*β* and IL-18. (f) The intensity of each protein was normalized to the signal intensity of the corresponding pro-IL-1*β* or pro-IL-18. *n* = 6. ^*∗∗*^*P* < 0.01 compared to the sham group. ^##^*P* < 0.01 compared to the CCI group.

To explore the potential molecular mechanism by which miR-223 may affect neuropathic pain, as shown in Figure 3(a), we predict the base binding site between wild-type/mutant miR-223 and NLRP3 using starBase (http://starbase.sysu.edu.cn). To confirm the interaction between NLRP3 and miR-223, we cotransfected BV2 cells with miR-223 mimetics or controls (NC) and dual-luciferase vectors containing wt or mut NLRP3. The luciferase reporter gene test showed that miR-223 significantly reduced the luciferase activity of wt NLRP3 but did not reduce the luciferase activity of mut NLRP3, so it was confirmed that NLRP3 is the target gene of miR-223 (Figure 3(b)). Next, on the 7th day after surgery, Western blot analysis was used to examine the NLRP3, ASC, and activated caspase-1 in the spinal cord of CCI model mice infected with Lenti-miR-223 or control Lenti-vector. Relative protein levels are shown in Figure 3(c). The results showed that the levels of NLRP3, ASC, and activated caspase-1 protein in the spinal cord of CCI mice were significantly higher than those of sham-operated mice. Moreover, the overexpression of miR-223 significantly suppressed the expression levels of NLRP3, ASC, and activated caspase-1 in the spinal cord compared to the control CCI treatment (Figure 3(d)). Further research revealed protein levels of mature IL-1*β* and IL-18 in the spinal cord (Figure 3(e)). As shown in Figure 3(f), the protein levels of the two cytokines in CCI mice are higher than that of sham mice, and the overexpression of miR-223 reduces the maturation of cytokines. These findings further confirm the interaction between miR-223 and NLRP3 and suggest that miR-223 downregulates the expression of NLRP3, thereby inhibiting the neuroinflammatory activity.

### 3.4. The Effect of miR-223 Inhibition on Macrophage Subsets in Injured Peripheral Nerves

**Figure 4:**
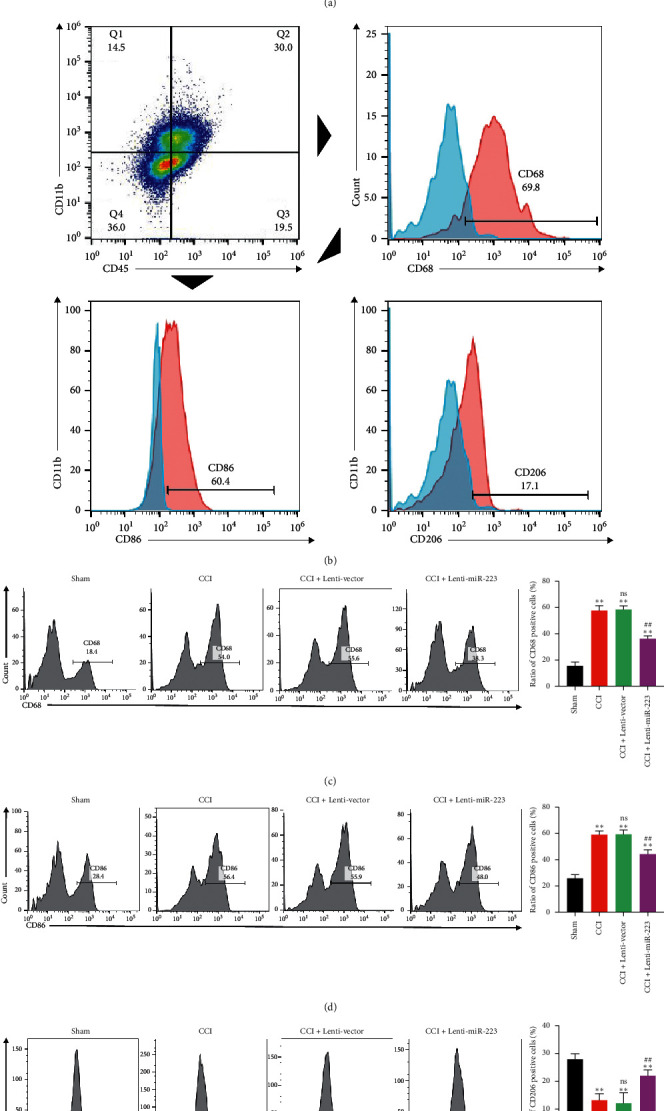
The effect of miR-223 inhibition on macrophage subsets in injured peripheral nerves. (a) Chemotaxis experiment of RAW264.7 and glial cells derived from mice injected with Lenti-vector or Lenti-miR-223. Cell count staining showed that LPS-treated microglial cells had a significant recruitment effect on RAW264.7 cells, while overexpression of miR-223 significantly reduced the recruitment effect. (b) Cells were isolated from peripheral nerves, and then, macrophages were selected based on the expression of CD45 and CD11b. Among CD45+ and CD11b + macrophages, they were analyzed for the expression of CD86 and CD206. (c) Compared with the sham group, the total number of macrophages in each group was significantly increased, while the overexpression of miR-223 significantly inhibited the recruitment of macrophages. *n* = 6. ^*∗∗*^*P* < 0.01, a significant statistical difference compared with the sham group. ns, no significant statistical difference compared with the CCI group. ^##^*P* < 0.01, a significant statistical difference compared with the CCI + Lenti-vector group. (d) The number of CD86 positive cells in each group. CD86 positive cells in the CCI group increased significantly. miR-223 overexpression significantly reduced the number of CD86 positive cells. *n* = 6. ^*∗∗*^*P* < 0.01, a significant statistical difference compared with the sham group. ns, no significant statistical difference compared with the CCI group. ^##^*P* < 0.01, a significant statistical difference compared with the CCI + Lenti-vector group. (e) The number of CD206 positive cells in each group; compared to the CCI group, overexpression of miR-223 significantly increased the number of CD206+ positive macrophages cells. *n* = 6. ^*∗∗*^*P* < 0.01, a significant statistical difference compared with the sham group; ns, no significant statistical difference compare with the CCI group. ^##^*P* < 0.01, a significant statistical difference compared with the CCI + Lenti-vector group.

To further examine the effect of miR-223 on neuroinflammation, we tested the recruitment of miR-223 on macrophages in glial cells in vitro. As shown in Figure 4(a), microglial cells were isolated from mice injected with Lenti-vector or Lenti-miR-223, and transwell was used for RAW264.7 macrophage chemotaxis experiment. Data show that RAW264.7 in the LPS + Lenti-vector group significantly recruited to another side and the miR-223 overexpression significantly inhibited the recruitment phenomenon.

By using flow cytometry analysis, nerve macrophages were identified as CD45 and CD11b expressing cells [16]. Among them, macrophage subsets were analyzed by the expression of CD86 and/or CD206. As shown in Figure 4(b), cells isolated from sciatic nerves were first analyzed for leukocyte common antigen CD45 and integrin alpha-M beta-2 CD11b expression. Among them, mark the total amount of macrophages with CD68. CD86 provides costimulatory signals necessary for T cell activation, considered a cell surface marker for M1 macrophages. CD206 is a mannose receptor implicated in endocytosis. It is a marker specifically associated with M2 macrophages. The total number of macrophages expressing CD86 or CD206 was quantified by flow cytometry. Compared with the sham group, the total number of macrophages increased significantly in the CCI and CCI + Lenti-vector groups, while the total number of macrophages in the CCI + Lenti-miR-223 group markedly decreased (Figure 4(c)). For the M1 macrophages, CD86+ macrophages cells in CCI were significantly increased, while overexpression of miR-223 significantly reduced the number of CD86+ positive macrophages cells (Figure 4(d)). They were indicating that miR-233 inhibits the damage effects to the nerve induced by macrophages. For the M2 macrophages, compared to the CCI group, overexpression of miR-223 significantly increased the number of CD206+ positive macrophage cells (Figure 4(e)). Indicating the protective effect of M2 macrophages on nerves could be activated by miR-223.

## 4. Discussion

Neuropathic pain is chronic pain. It is the most severe type of chronic pain and can cause a substantial public health burden. The current study found that miR-223 can reduce the expression of NLRP3, ASC, caspase-1, IL-1*β*, and IL-18 in CCI mice. Inflammasomes contain high molecular weight signaling platforms that resulted in the activation of inflammatory caspases. Activated caspase-1 plays a vital role in the proteolytic processing and maturation of the cytokine precursors of IL-1*β* and IL-18. NLRP family proteins can quickly recognize the inflammation induced by pathogens and participate in the processing of IL-1*β* and IL-18 [17].

Our research shows that overexpression of miR-223 in mice inhibits the production of IL-1*β* by inflammatory bodies by inhibiting the expression of NLRP3 protein. Similar to our current study, recent studies have indicated that after stroke, miR-223 can downregulate NLRP3 to negatively regulate caspase-1 and IL-1*β* to suppress neuroinflammation [18]. NLRP3 controls caspase-1 activation by combining with ASC containing caspase recruitment domain and cysteine-aspartic protease (caspase) to form oligomers. Caspase-1 is invertase of IL-1*β*; activated caspase-1 can promote the maturation of IL-1*β* and secrete it out of cells to participate in the inflammatory response. It has been reported that release of IL-1*β*-mediated CFA-induced pain hypersensitivity and IL-1*β* contribute to pain signal [19]. It has been reported that activation of AMPK inhibits NF-kB activation and IL-1*β* expression and contributes to the analgesic effect [20]. Also, NLPR3 can activate NF-*κ*B, thereby starting the transcription of IL-1*β* precursor [21].

Our research shows that NLRP3 is upregulated and plays a role in a mice model of sciatic nerve ligation pain. Similarly, the chemotherapy-induced neuropathic pain neuropathy model showed that both the oxaliplatin-induced neural injury model and the paclitaxel-induced neural injury model were upregulated, while inhibition of NLRP3 reduced mechanical pain-like behavior [22]. However, research indicates that different neuropathic pain models (sparse nerve injury, spinal nerve transection, spinal nerve ligation, and partial nerve ligation) will not produce the same findings [23].

Macrophages and microglia are the most abundant immune cells activated in injured nerves and the spinal cord, respectively [24]. Several pieces of evidence indicate that the survival, activation, proliferation, and differentiation of macrophages/microglia require the participation of macrophage colony-stimulating factors. In this study, we investigated whether blocking macrophage colony-stimulating factor/colony-stimulating factor 1 receptor signaling can effectively relieve neuropathic pain.

Macrophages can be divided into classically activated M1-like proinflammatory and alternately activated M2-like anti-inflammatory or wound healing phenotypes. After polarization/activation, they do express different cell markers, including receptors, enzymes, trophic factors, chemokines, and cytokines [25]. Generally, M1 macrophages are considered neurotoxic, while M2 macrophages promote the regeneration of damaged axons. According to the report, M1-like macrophage strongly expresses inflammatory cytokines that may be responsible for neuropathic pain [9], while the M2 phenotype has enhanced anti-inflammatory properties, and it has been shown that M1-type macrophages are involved in hyperexcitability and pain-related substances [26]. In this study, we used M1-related markers, one of CD86 and M2 related, to study the effect of miR-223 overexpression on macrophage subsets. In the injured sciatic nerve, CD45 and CD11b-labeled macrophages are labeled with CD206. In this study, we observed that the inhibitory effect of miR-223 overexpression on NLRP3 could significantly reduce the number of macrophages, enhance M2-like macrophages, and inhibit M1-like macrophages. This indicates that miR-223 has a protective effect on macrophage-mediated neuroinflammatory damage by downregulating NLRP3. However, whether blocking NLRP3 signaling can enhance anti-inflammatory properties while reducing proinflammatory macrophages in damaged nerves require further study.

## Data Availability

The raw/processed data required to reproduce these findings cannot be shared as the data also form part of an ongoing study.
