# Erratum to “miR-223 Inhibits the Polarization and Recruitment of Macrophages via NLRP3/IL-1*β* Pathway to Meliorate Neuropathic Pain”

**DOI:** 10.1155/2023/9756947

**Published:** 2023-03-09

**Authors:** Junsong Zhu, Jinmei Yang, Jianguo Xu

**Affiliations:** ^1^Pain Department, Puren Hospital Affiliated to Wuhan University of Science and Technology, Wuhan 430081, China; ^2^Wuhan Hospital of Traditional Chinese and Western Medicine, Wuhan 430022, China; ^3^Hubei Provincial Hospital of Traditional Chinese Medicine, Wuhan 430061, China; ^4^Hubei Provincial Academy of Traditional Chinese Medicine, Wuhan 430074, China

In the article titled “miR-223 Inhibits the Polarization and Recruitment of Macrophages via NLRP3/IL-1*β* Pathway to Meliorate Neuropathic Pain” [1], there was an error in the corresponding author details during production. The correct contact information is shown above.

## References

[1] Zhu J., Yang J., Xu J. (2021). miR-223 Inhibits the Polarization and Recruitment of Macrophages via NLRP3/IL-1*β* Pathway to Meliorate Neuropathic Pain. *Pain Research and Management*.

